# Compulsory treatment at home: an interview study exploring the experiences of an early group of patients, relatives and mental-health workers

**DOI:** 10.1186/s12913-024-11787-2

**Published:** 2024-11-05

**Authors:** D. A. de Waardt, I. C. de Jong, M. Lubben, I. Haakma, C. L. Mulder, G. A. M. Widdershoven

**Affiliations:** 1 Department of Psychiatry, ETZ Hospital (Elisabeth-TweeSteden Ziekenhuis), Tilburg, the Netherlands; 2 Department of Ethics, Law and Humanities, Amsterdam University Medical Centers (location VUmc), Vrije Universiteit Amsterdam, Amsterdam, the Netherlands; 3 GGZ VS, Utrecht, the Netherlands; 4 Lentis Research, Lentis, Groningen, the Netherlands; 5 Parnassia Psychiatric Institute, Rotterdam, the Netherlands; 6 Department of Psychiatry, Epidemiological and Social Psychiatric Research institute (ESPRi), Erasmus University Medical Center, Rotterdam, the Netherlands

**Keywords:** Mental health legislation, Compulsory Community Treatment, Compulsory Treatment in patients' Homes, Compulsory mental health care, Stakeholders' experience

## Abstract

**Background:**

When introduced in 2020, the Netherlands’ Compulsory Mental Healthcare Act included provisions for compulsory community treatment (CCT) and compulsory treatment in patients’ homes (CTH). Although CCT has been incorporated into mental health care in many countries, its effectiveness is debated. We know of no other countries in which CTH has been adopted.

The aim of this study is to evaluate how an early group of participants experienced CTH. They were drawn from three stakeholder groups: patients, relatives and mental-health workers.

**Methods:**

In total, 17 open interviews were conducted with six patients, five relatives and six mental-health workers. All had experience with CTH. Thematic analysis was used to analyze the interviews.

**Results:**

Five themes were identified: 1). Reasons for applying for a court order with options for CTH. The reasons included preventing harm, avoiding hospitalization, and providing a safety net. 2.) Participants’ experiences with CTH in practice. The four most noteworthy experiences were related to the process of applying for a court order; compulsory home visits and the compulsory use of medication; the involvement of relatives during treatment; and the influence of CTH on the relationship between patients and relatives.  3.) The advantages and disadvantages of CTH. The most important advantages were avoiding hospitalization; improving medication adherence; facilitating easy access to care; early signaling of deterioration; early intervention; and regained autonomy. The most important disadvantages were restricted autonomy; fewer options for monitoring compared to hospitalization; and problems regarding control of patient behavior. 4.) Participants’ preferences. All preferred CTH to hospitalization. 5.) Participants’ suggestions for improving CTH. These included the need not only to provide patients with better information, but also to improve the involvement of relatives during treatment.

**Conclusion:**

The interviewees found that CTH might help to avoid hospitalization by providing stakeholders with more options for arranging effective care at home. Although this suggests that initial experiences of CTH under the new Dutch mental health law were positive, it is still uncertain whether CTH as currently implemented really differs from CCT.

**Supplementary Information:**

The online version contains supplementary material available at 10.1186/s12913-024-11787-2.

## Introduction

The Compulsory Mental Healthcare Act, which was introduced in the Netherlands on January 1, 2020, provides for the use of compulsory community treatment (CCT) and for compulsory treatment in the patient’s home (CTH).

CCT, which has been implemented in mental health care in many countries [1], obliges patients who are under a court order to comply with certain treatment conditions outside of an institution. Any patient who fails to meet these conditions can be readmitted to a psychiatric hospital.

Unlike CCT, CTH has not, to the best of our knowledge, been implemented elsewhere in the world. While CCT means that patients who do not comply with their treatment conditions can be readmitted, it is implicit that CTH may allow mental-health workers to use compulsion in patients’ homes, such as the use of physical force to ensure that they take their medication.

The effectiveness of CCT has been much debated. No conclusive evidence has been found in Randomized Controlled Trials and pre- and post-studies that it is more effective than voluntary care in improving clinical outcomes, reducing the number of hospitalizations, or reducing the time spent in hospital [2–4]. As well as clinical outcome measures, several studies have investigated stakeholders’ views and preferences regarding compulsory care in the community. In a large-scale constant comparative analysis of published qualitative research on three stakeholder groups’ opinions of CCT, Corring et al. [5] found that patients, relatives, and mental-health workers all believed the benefits of CCT to outweigh its coercive nature. An integrative review of stakeholders’ opinions also found that a majority supported its use, even though the stakeholders had concerns regarding stigmatization, a focus on medication, and the restrictions CCT imposed on patients’ freedom and autonomy [6].

CTH was introduced in the Netherlands as an addition to CCT. In theory, it allows people to be treated against their will in their own homes, and also means that they, for example, could be forced to live in sheltered accommodation.

Its use is relatively new. Although several studies have investigated the effects of CCT and people’s experiences with it, none that we know of have studied the effects of CTH or the experiences with CTH. We therefore investigated how CTH was experienced by three stakeholder groups: patients, relatives and mental-health workers. Our results are intended to help improving the practice of mental health care in the community.

## Methods

### Setting

#### Compulsory care as regulated and defined in the mental health law in the Netherlands

Involuntary care in the Netherlands begins with a court order issued by a public prosecutor on the basis of a report by a psychiatrist who is in charge of involuntary care at a mental healthcare institution. The report itself is based both on the care plan drawn up by the mental-health workers involved in the patient’s care and on the second opinion provided by an independent psychiatrist.

The care plan and second opinion must both specify the types of involuntary care being applied for. The Mental Health Act describes eleven types of compulsory care. As Table 1 shows, these range from compulsory medication through compulsory house-searches (for the presence of drugs, for example) to hospitalization, which is seen as a last resort. Other forms of compulsory care, such as medication, blood tests, or regular appointments with the mental health team, can also be ordered for patients living at home.

**Table 1:**
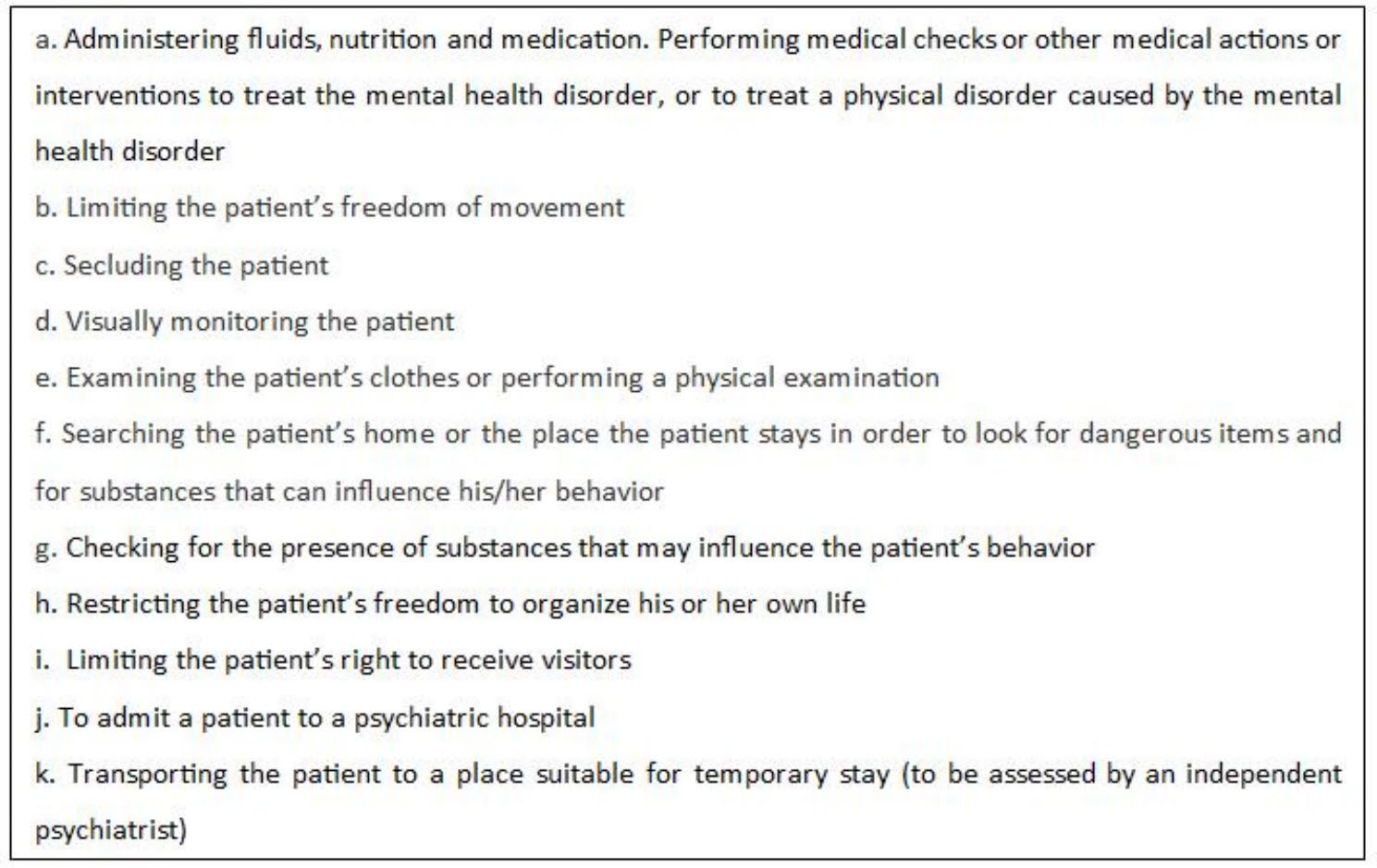
The forms of potential involuntary treatment that, under the definitions of the Dutch Compulsory Mental Healthcare Act, can be applied inside or outside the hospital, and thus at the patients’ home

Before deciding to issue a court order, the judge holds a hearing that is attended by the patient, the treating psychiatrist, and sometimes by the public prosecutor [7]. When a judge decides to issue a court order, the judge also decides which forms of compulsory treatment will be allowed. This does not mean that all the forms of compulsory care specified will eventually be used in practice; it is for the psychiatrist or clinical psychologist who treats the patient to decide which forms are necessary.

### Participants

Patients were recruited from the outpatient teams at two large mental healthcare organizations, one in the northern Netherlands and one in the southern Netherlands. After selection, they were informed about the study by their mental-health workers in these community teams. If they agreed to be interviewed, they signed the informed consent form and were asked whether we could approach one of their relatives and their mental-health worker for an interview. The relatives and mental-health workers in question were informed, and also signed an informed consent form.

### Interviews and analysis

The interviews were carried out by DW and ML between May 2020 and September 2021. They were held either face to face, or, due to covid restrictions, by phone. These semi-structured interviews, which were based on a topic list with several questions, were recorded and transcribed verbatim. The interviews were designed for this study and are available as supplementary material.

Thematic analysis [8] was used to analyze the data from the interviews. The data provided by the patients, relatives and mental-health workers was combined into a single analysis.

After independently analyzing the same seven interviews, DW and IdJ compared the codes they had found until they reached consensus over each code and quotation. Next, the codes were clustered by DW, IdJ and GW. This resulted in five main themes, each with subthemes.

The remaining ten interviews were used to expand the dataset and to see whether new themes emerged, which was not the case. The thematic analysis was performed using Atlas.ti.

## Results

Seventeen respondents were interviewed: six patients, five relatives and six mental-health workers. Four patients were male and two were female; they had been diagnosed with bipolar disorder, schizophrenia or schizoaffective disorder. Different family members participated: two mothers, one sister, one son and one daughter. One patient could not name a relative who could participate. Five of the patients’ mental health care workers were community nurses and one was a psychiatrist.

For the patients in this study, compulsory treatment at home consisted of obligatory home visits, in some cases accompanied by the administration of intramuscular depot injection or by giving medication to be taken orally. For one patient, this meant that he was told where he had to live.

**Fig. 1:**
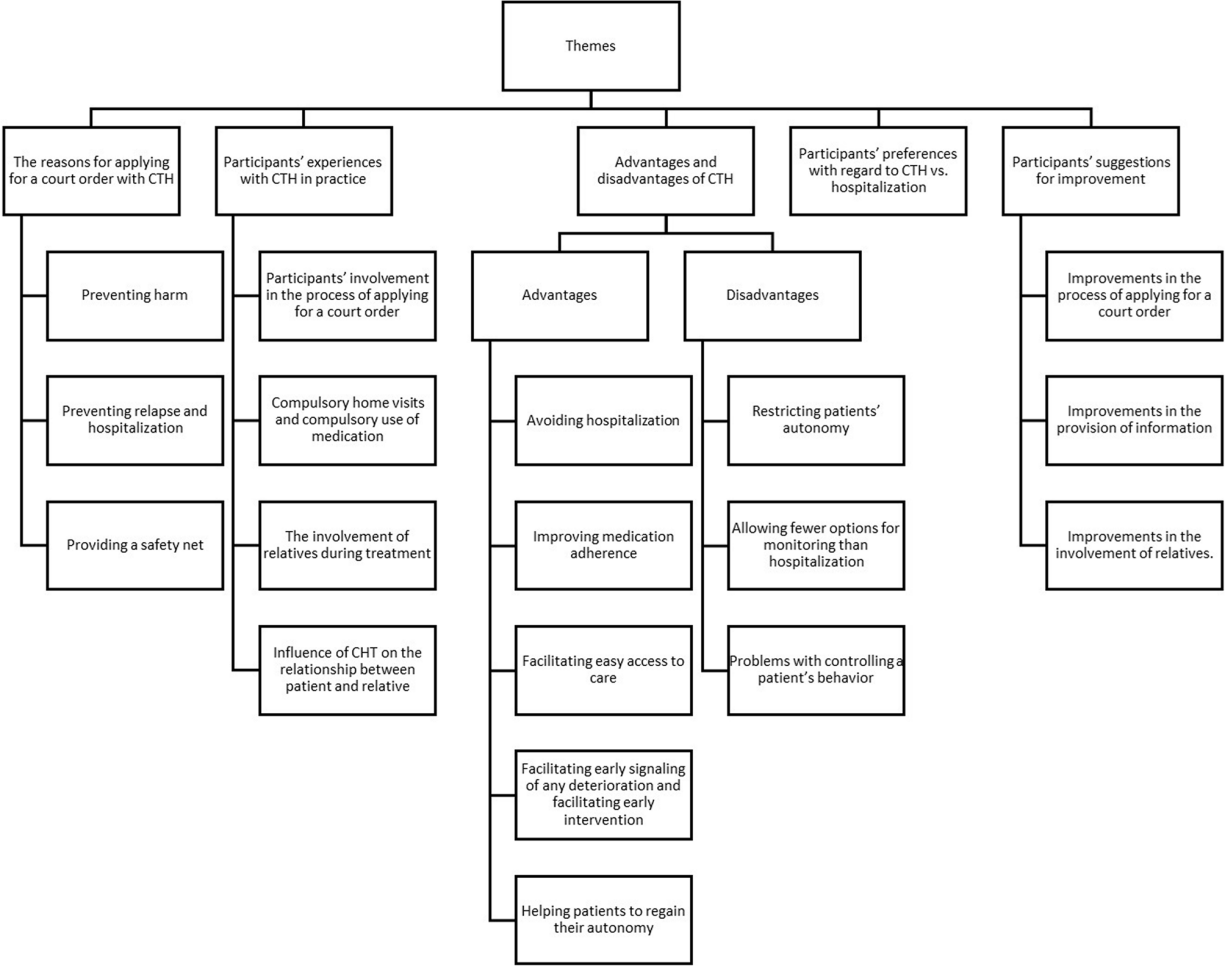
Themes and subthemes

Analysis of the interviews identified five themes: the reasons for applying for a court order with options for CTH, participants’ experiences with CTH in practice, the advantages and disadvantages of CTH, participants’ preferences with regard to CTH vs. hospitalization, and their suggestions for improving CTH. The five themes and their subthemes are discussed below and are summarized in the coding tree (Fig. 1).

## The reasons for applying for a court order with CTH

Three main reasons for applying for a court order with CTH were found: (1) to prevent harm, (2) to prevent relapse and subsequent hospitalization, and (3) to provide a safety net.

### Preventing harm

The main reason for applying for a court order reported by patients, relatives and professionals was the need to prevent harm to the patient and others. This referred to various kinds of harm, such as aggression, spending too much money, or self-neglect and was mentioned by patients, relatives and professionals. For example, the following statement was made by the relative of a patient who had run a risk of serious illness when she stopped eating during an earlier psychotic episode:

*“She was psychotic*,* had delusions*,* was paranoid. She constantly ran away and roamed the streets. She stopped eating because she was frightened of being poisoned.” (relative)*

A patient stated that he could become aggressive when he was unwell – something he wished to prevent:

*“the court order was intended to prevent me from becoming aggressive*,* hyperactive and unrealistic. That was the main thing – to protect me from that.” (patient)*

One mental health worker mentioned that his patient did not stay in his house but lived in the woods when he became ill and did not take care of himself.

*“Because*,* you see*,* he ate very little*,* he only ate something when he visited his mother*,* so there was risk he would starve or freeze to death outside’’. (mental health worker)*

### Preventing relapse and hospitalization

Many participants gave as reason for a court order with CTH: to prevent the mental and physical health of the patient from deteriorating, and to avoid hospitalization.

One patient stated that she needs health care providers to intervene rapidly with medication when she becomes manic. Without such intervention, her situation would quickly deteriorate.

*“So it has to be very quick. Because if it isn’t done right away*,* it’s like an avalanche – it all goes very bad.” (patient)*

One relative explained that when the patient got manic and they intervened early, they could prevent admission to hospital.

*''He suffered from manic episodes*,* and when we would recognize in an early phase that he was getting manic again and he would get his medication*,* we could turn the situation around.’’ (relative)*

### Providing a safety net

Most patients, relatives and professionals stated that the court order served as a safety net that enabled them to monitor the patient, keep in touch and facilitate access to care when he or she was not doing well.

*''For me*,* it’s like a necessary evil: I need a court order as a safety precaution.’’ (patient)*

*''She says that it’s good to have some sort of big stick*,* both for herself and for us.’’ (relative)*

*“And then you see all these signs – you can see he’s not doing well*,* so we try to monitor closely*,* just in case.’’ (mental health worker)*

Patients and relatives also saw the CTH court order as a means of facilitating access to hospital if admission was unexpectedly deemed necessary. The law allows patients who already have such an order to be admitted immediately if their mental health deteriorates, without the need to apply for a new legal procedure.

## Participants’ experiences with CTH in practice

Participants’ descriptions of their experiences concerned four subthemes: (1) their involvement in the process of applying for a court order with CTH; (2) compulsory home visits and the compulsory use of medication; (3) the involvement of relatives during treatment; and (4) the influence of CTH on the relationship between patients and their relatives.

### Participants’ involvement in the process of applying for a court order

When looking back on the process of applying for a court order, patients and relatives reported their involvement differently. Most patients said they did not understand what was going on at the time of application, sometimes because they were too ill or because they did not want to know about the court order.

*“There was a video conference with the judge and my lawyer. And I was there with the psychiatrist and his assistant*,* but I can’t remember anything. I know I got a court order for six months*,* but I only know that because my sister told me. I can’t remember anything.” (patient)*

In contrast, most family members did indeed feel they were involved in the process of applying for a court order, and expressed their satisfaction about it .

*“Together – the home care worker*,* the community nurse*,* the general practitioner and me – we decided to apply for a court order. It was a lot of paperwork*,* but it turned out well.” (relative)*

### Compulsory home visits and compulsory use of medication

In all cases, the court ordered obligatory home visits by mental-health workers. Most patients said that the contact with their mental health care workers was good, and that they did not experience home visits as compulsory care.

*“I think I see her often enough. She comes once every four weeks*,* and if something is the matter*,* I can always call.” (patient)*

*''What do I think of these home visits? They’re a good thing*,* a beautiful thing.’’ (patient)*

However, one patient stated that he did not want the psychiatrist to visit him *''Yes*,* he’s been here once. But that was the last time – I don’t want him here again.’’ (patient)*

Opinions regarding the compulsory use of medication varied. Some patients, relatives and mental-health workers were positive about its use:

*“I think it’s a good thing. I receive depot medication only once every four weeks*,* and have been doing well with it since I came home.” (patient)*

*“To me*,* it is a good thing that he receives an injection*,* now he does not have to take any more medication.’’ (relative)*

*“The depot medication really helped him to stabilize and to remain stable” (mental health worker)*

One patient agreed that medication helped him, but that he wanted to lower the dose.

Another patient was clearly opposed to the use of medication: *‘’Do medicines help? Take them yourself!’’ (patient)*

Patients and relatives also referred to the side-effects of medication, mainly their sedative effect:

*“You’re influenced by the medication – like 60*,* 50*,* 40%. You’re heavily influenced.’’ (patient)*

*“And then I called them again and said: ‘I’ fear the medication is making her less steady on her feet.’ I know what this medication can do. Sometimes you have to choose between two bad options.’’ (relative)*

### The involvement of relatives during treatment

One aim of the new mental health law in the Netherlands is to increase relatives’ involvement in the application of compulsory care. Most relatives and patients stated that relatives were involved in the treatment. The role of the relatives consisted mainly of supporting the patient and signaling any deterioration in their mental health.

*“Soon we will have an evaluation with my family about what happened last year and how everything went. My sisters will be there and that is a good thing.’’ (patient)*

*“Yes*,* we are very much involved. If something is the matter*,* we are the first to be in touch [with the mental health care team]''. (relative)*

On the other hand, relatives felt that they should have been better informed, and that greater attention should be paid to their opinions.

*“Yes*,* we can be important and would like to be heard – especially at the beginning of a new treatment plan or when there are important milestones.'' (relative)*.

The healthcare workers were all very positive about the role of the relatives:

*“This family is very involved. At first it was his parents*,* but once they got older*,* his sister took over their role.’’ (mental health worker)*

*“Well*,* at one point we decided to make this choice for him*,* but we did so in consultation with his psychiatrist and his mother.” (mental health worker)*

### Influence of CHT on the relationship between patient and relative

Overall, relatives and patients did not mention any negative effects of CTH on their relationship.

*''When I’m stable*,* it doesn’t have any consequences [for our relationship].’’ (patient)*.

*''No*,* the treatment didn’t change our relationship.” (relative)*.

However, one relative stated that she was very careful not to interfere with the treatment, and to keep her role strictly to that of a daughter, not that of a mental-health worker, as she did not want to mix these roles out of a fear influencing the relationship with her mother:

*“I don’t want to get involved like that. I can’t*,* because I’m her daughter – I’m emotionally attached and very worried. I want her to get better. A psychiatrist looks at these things from a different perspective.” (relative)*

## Advantages and disadvantages of CTH

The participants indicated several advantages and disadvantages of CTH.

### Advantages of CTH

Participants expected that CTH would facilitate early intervention in a patient’s house, and thereby enable them to stay at home. Relatives and mental-health workers in particular regarded the possibility of avoiding hospitalization as an important advantage:

*“With the right medication*,* I think she can get some peace in her head*,* and can remain living at home.” (relative)*

*“I think the principle of CTH is good. If people aren’t taken out of their home environment*,* I can imagine that they recover faster and easier.’’ (relative)*

A mental-health worker said CTH could prevent compulsory hospitalization. *“Yes*,* it’s a great advantage that you can save someone from hospitalization and can provide certain parts of the treatment outside the hospital.” (mental health worker)*

Some participants stated that CTH increased medication adherence. One reason was that patients were less likely to forget it. Another was that they had to accept depot medication:

*“Now I have this depot*,* I can’t forget my medication.” (patient)*

*“He would have never taken the depot if he hadn’t had a court order. So in that respect*,* I think it’s a great advantage. Without a court order*,* I don’t think we’d have gotten there any other way.’’ (mental health worker)*

However, one mental-health worker doubted whether providing medication at home actually improved adherence:

*“I very much doubt whether she takes [her medication]. But I keep on taking it to her.” (mental health worker)*

Patients and relatives also reported that CTH provided easy access to care, especially when hospitalization turned out to be necessary. Once someone has a court order specifying that hospitalization is a possible form of compulsory care, no extra procedures are required if hospitalization seems necessary:

*“Yes*,* I like it that everything has been arranged. It is not nice*,* having to arrange everything the moment it goes wrong. I know from experience that the mayor has to come ….while everything is going round in my head and then I get so upset.’’ (patient)*

*“For us*, *that’s mainly the power of the court order*, *so to speak – that if something happens*, *we know we won’t have to keep her indoors at all costs while waiting to go through all the necessary procedures.’’ (relative)*

Another advantage that was often mentioned is the option for early warning and early intervention if a patient’s condition deteriorated. Relatives and mental-health workers said they were positive about CTH’s potential for closer monitoring at home and for earlier intervention than would be possible for patients in voluntary care.

*“When you can monitor very closely*,* you can see certain signs*,* such as when he didn’t open his post.” (mental health worker)*

Finally, participants reported that CTH could sometimes help patients to regain their autonomy. If their condition was treated, and if they were provided with what they needed (such as proper housing or someone to help monitor finances), they might be able to take control of their lives:

*“As he has no insight into his disorder*,* he’s unable to take control of his own life without a court order. Without one*,* he’ll refuse medication and will get unsettled.” (mental health worker)*

### Disadvantages of CTH

Although the idea that CTH can foster autonomy was mentioned as an advantage, it was sometimes felt that CTH could restrict it. One patient reported that he was not allowed to choose where to live; several patients stated they viewed the obligatory use of medication as an infringement of their autonomy:

*“In the sense that I’m less allowed to do things […] I am not allowed to move*,* I would not do that immediately anyway*,* but [I wish] I was allowed to*,* or to buy a house.” (patient)*

*“Well*,* I’m restricted in things I’d like to do.’’ (patient)*

The mental-health workers all referred to the restrictive nature of the CTH:

*“I think it’s a big thing that we take over certain things from people because of this compulsory care.” (mental- health worker)*

Another disadvantage of CTH regarded monitoring. Relatives said that it was hard to know how a patient was doing when they were at home alone. Some reported that CTH provided fewer options for monitoring a patient than if they had been hospitalized:

*“If no-one else is there*,* yes*,* they could easily pretend to be fine on the phone*,* and if no-one is in the position to visit*,* like last year*,* a situation could get out of hand.” (relative)*

*“And I’ve already told the community nurse that I’d really like to have a camera installed there*,* but that’s not allowed.” (relative)*

A final disadvantage, which was raised mainly by mental-health workers, concerned problems regarding the control of patient behavior. For example, it was hard to know whether someone was actually taking their oral medication. Similarly, a patient could not be forced to actually take their oral medication, or it was hard to keep track of someone who did not come to appointments:

*“The disadvantage lies in actually providing compulsory care. I find that very complicated*,* especially when it comes to oral medication*,* for example. When people really don’t want to take medication*,* it’s hard to provide it responsibly at home.” (mental health worker)*

## Participants’ preferences with regard to CTH vs. hospitalization

Despite the concerns and disadvantages, all participants preferred – under the right conditions – CTH to involuntary hospitalization:

*“Interviewer: And what would you prefer? An injection at home and staying at home*,* or hospitalization? Patient: At home of course!” (patient)*

*''Overall*,* if the right conditions could be created for delivering compulsory care at home*,* I think it would be a great advantage.” (relative)*

*“If the situation at home is all right*,* I’d prefer [care] at home!” (mental health worker)*

## Participants’ suggestions for improvement

The participants had three suggestions for improving CTH: (1) improvements in the process of applying for a court order, (2) in the provision of information, and (3) in the involvement of relatives.

### Applying for a court order

Participants found the application procedure for a court order to be complex, especially those who were mentally ill when the application was made. Mental-health workers reported having to fill out a lot of paperwork, and suggested that this be reduced:

*“Yes*,* the process could be improved. There are some very complex documents*,* both for the mental-health workers and for the patients. [….] While it all seems to have been put together very carefully*,* the process is so complex that it makes you pay less attention to the actual content.” (mental health worker)*

A patient suggested extending the period for which a court order is valid: he experienced the renewal process as painful and stressful, and did not want to go through it every year:

*“Whenever I have to think about it again*,* I get a lump in my throat. It’s such an awful time*,* always. When we have to apply for a new court order*,* a lot comes up*,* just like that. It shows how vulnerable you are.” (patient)*

A healthcare worker had a similar experience in another case:

*“What bothers him is that he has to be present at the hearing to renew the order. I promised him that I’d go to court with him. And that I’d give him a sedative*,* because he really dreads it.” (mental health worker)*

### Provision of information

Overall, patients, relatives and mental-health workers all referred to the need for patients and relatives to be provided with better information, with regard both to the legal procedures and to developments in the treatment process:

*“What I needed? To be kept up to date by the people who treated my son. That would have given me peace of mind.’’ (relative)*

*“Improving the process would also mean improving that paperwork.’’ (mental health worker)*

One patient said that he would have liked to be informed again about the legal procedures when he was doing better.

*''When I’m ill*,* I don’t realize all these things. But once I get better*,* that’s when they should discuss them with me.’’ (patient)*

Another patient said that she did not understand the legal documents:

*“Well*,* actually*,* not really. I don’t really understand the language.” (patient)*

Mental health workers also stated that it should be made clearer to patients what kind of compulsion might actually take place in their home, and in which situations they would be hospitalized. A healthcare worker, referring to the current policy of the Dutch Psychiatric Association not to use compulsion when a patient physically resists, stated that such information should be communicated to patients:

*“We’re really very reluctant to use compulsion at home*,* although in theory it’s possible that we’ll use it. […] I’d like to [explain to patients] that we won’t use it. [….]. Otherwise*,* when people start reading these documents*,* they’ll think that these things can happen [to them].’’ (mental-health worker)*

### Involvement of relatives

Relatives suggested that more attention should be paid to their involvement during treatment. They said that because they knew the patient’s history and saw their functioning on a daily basis, they could contribute more to the treatment than was currently possible:

*“Because of our experiences in the past*,* I think we have a lot of knowledge. But they don’t ask us or act upon our experiences. (relative)*

*“It’s all very distant right now*,* you know. The community nurse comes around for a cup of coffee every now and then. But he doesn’t always have the time – he’s very busy. I hardly get involved. […] I hardly hear from them.” (relative)*

## Discussion

This study explored how patients, relatives and mental-health workers had experienced compulsory treatment at home (CTH). To our knowledge, this was the first time such stakeholders have been questioned on this new form of compulsory community treatment (CCT).

The most important reasons participants gave for applying for court-ordered CTH were to prevent harm, to avoid hospitalization, and to provide a safety net. These reasons are comparable to those identified in earlier studies as advantages of CCT [6]. With regard to avoiding hospitalization, however, studies on the effect of CCT did not demonstrate that CCT was more effective than voluntary outpatient care [2, 3]. Even so, on an individual level, it might be the case that CCT and CTH help a specific subgroup of patients to avoid hospitalization.

Overall, the advantages and disadvantages of CTH we found in our study were consistent with the advantages and disadvantages of CCT found in earlier studies. A difference is that studies on CCT found overemphasis on medication to be a disadvantage [9–12]. Although oral and intramuscular medication were also important treatment conditions in our study, our participants did not refer to a primary focus on medication. On the contrary: some participants – patients included – stated that medication helped patients to regain stability and to foster autonomy in the sense of having control over their lives.

Since the advantages and disadvantages of CCT and CTH seem to be so similar, one might wonder whether, in practice, CTH really differs from its counterpart CCT, which has previously been used in various countries, including the Netherlands. While, in theory, CTH allows for the use of several forms of compulsory treatment at home (Table 1), the actual use of compulsion is often limited, given that the Dutch Psychiatric Association advises against compulsion when a patient physically resists treatment or other coercive measures [13]. In such cases, hospitalization is proposed, as it is in other jurisdictions when treating people with CCT.

Our finding that, overall, the participants in our study preferred CTH to compulsory hospitalization contrasts with findings from an earlier study in which one third of the patients and one third of the relatives in a large cohort preferred hospitalization to treatment at home [14]. One possible reason for this difference is that the patients and relatives in the earlier study had no experience with CTH, while the participants in our study had this experience, and were largely positive about it.

When interpreting studies on stakeholders’ opinions on CTH one should take into account the differences between comparing CTH with involuntary hospitalization on the one hand and with voluntary care in the community on the other hand. While a patient might prefer CTH to hospitalisation, he or she might choose voluntary care if given a choice between CTH and voluntary care in the community.

### Strengths

To our knowledge, this is the first study to evaluate stakeholders’ experiences of CTH from three perspectives. As we are unaware of any other country in which CTH has been implemented, it is important to evaluate what is potentially an invasive form of compulsory care.

### Limitations

Since participants were recruited through their mental-health workers, there may also have been a selection bias: the only participants to participate were those who were doing well enough and were also willing to do so.

Another limitation is the potential influence of bias during coding and analysis of the interviews. Regarding coding we made sure that each interview was coded twice, simultaneously by DW and IdJ to mitigate the risk of bias. Regarding analysis, we reflected on the potential influence of our background, having experience in clinical psychiatry (DW and NM) and psychiatric ethics (GW).

### Implications for future research

To improve CTH larger-scale studies should evaluate how CTH is used and how stakeholders experience it. Further research should identify the groups of patients that would benefit from CTH.

## Conclusion

Overall, the participants in this study were positive about the introduction of CTH. They preferred it to hospitalization, and reported that it potentially avoids hospitalization, facilitated access to care, and provided a safety net. Although such advantages are important, we should not overlook the disadvantages of using such a coercive measure. It still remains the question whether, in practice, the current use of CTH in the Netherlands differs from the application of CCT in other countries.

## Electronic supplementary material

Below is the link to the electronic supplementary material.

Supplementary Material 1.

## Data Availability

The interviews are not available due to privacy restrictions. However the results of the thematic analysis are available upon request.
