# Supplementary material for: Compulsory treatment at home: an interview study exploring the experiences of an early group of patients, relatives and mental-health workers
Source: BMC Health Serv Res. 2024 Nov 5;24:1346. doi: 10.1186/s12913-024-11787-2 (PMC11539426; doi:10.1186/s12913-024-11787-2)
Supplement: Supplementary file 1 — Supplementary Material 1. [file 12913_2024_11787_MOESM1_ESM.docx]

Interview guideline patient

1. Can you tell me about the process of applying for a court order?
2. Did you receive compulsory treatment at home?
3. If yes, what did you think about it?
4. Can you tell me about the advantages and disadvantages of compulsory treatment at home?
5. What do you prefer, compulsory treatment at home or admission to hospital?
6. Did the compulsory treatment affect your relationship with the mental health workers?
7. Did the compulsory treatment affect your relationship with your relatives?
8. Do you think the compulsory treatment at home will have an effect on you in the long term?

Interview guideline relative

1. Can you tell me about the process of applying for a court order?
2. Did your relative receive compulsory treatment at home?
3. If yes, what do you think about it?
4. Can you tell me about the advantages and disadvantages of compulsory treatment at home?
5. What do you prefer, compulsory treatment at home or admission to hospital?
6. Do you think the compulsory treatment influenced the relationship with the mental health workers?
7. Did your position towards your relative change because of the compulsory treatment at home?
8. Do you think the compulsory treatment at home will have an effect on you in the long term?

Interview guideline mental health worker

1. Can you tell me about the process of applying for a court order?
2. Did your patient receive compulsory treatment at home?
3. If yes, what do you think about it?
4. Can you tell me about the advantages and disadvantages of compulsory treatment at home?
5. What do you prefer, compulsory treatment at home or admission to hospital?
6. Do you think the compulsory care influenced the therapeutic relationship?
7. Do you think the compulsory treatment at home will have an effect on you in the long term?
